# Classification of FAMACHA© Scores with Support Vector Machine Algorithm from Body Condition Score and Hematological Parameters in Pelibuey Sheep

**DOI:** 10.3390/ani15050737

**Published:** 2025-03-04

**Authors:** Oswaldo Margarito Torres-Chable, Cem Tırınk, Rosa Inés Parra-Cortés, Miguel Ángel Gastelum Delgado, Ignacio Vázquez Martínez, Armando Gomez-Vazquez, Aldenamar Cruz-Hernandez, Enrique Camacho-Pérez, Dany Alejandro Dzib-Cauich, Uğur Şen, Hacer Tüfekci, Lütfi Bayyurt, Hilal Tozlu Çelik, Ömer Faruk Yılmaz, Alfonso J. Chay-Canul

**Affiliations:** 1 Division Académica de Ciencias Agropecuarias, Universidad Juárez Autónoma de Tabasco, Carr. Villahermosa-Teapa, km 25, Villahermosa CP 86280, Tabasco, Mexico; oswaldo.torres@ujat.mx (O.M.T.-C.); miguel.angel.gastelum@uas.edu.mx (M.Á.G.D.); ignacio.vazquez@correo.buap.mx (I.V.M.); armando.gomez@ujat.mx (A.G.-V.); aldenamar.cruz@ujat.mx (A.C.-H.); alfonso.chay@ujat.mx (A.J.C.-C.); 2 Department of Animal Science, Faculty of Agriculture, Igdir University, TR76000 Iğdır, Türkiye; 3 Área de Ciencias Agropecuarias, Grupo de Investigación en Ciencia Animal, Universidad de Ciencias Aplicadas y Ambientales U.D.C.A, Bogotá CP 111166, Colombia; rosparra@udca.edu.co; 4 Facultad de Ingeniería, Universidad Autónoma de Yucatán, Av. Industrias No Contaminantes s/n, Mérida CP 97203, Yucatán, Mexico; enrique.camacho@gmail.com; 5 Tecnológico Nacional de México, Instituto Tecnológico Superior de Calkiní, Av. Ah-Canul, Calkiní CP 24900, Campeche, Mexico; dadzib@itescam.edu.mx; 6 Department of Agricultural Biotechnology, Faculty of Agriculture, Ondokuz Mayis University, TR55139 Samsun, Türkiye; ugur.sen@omu.edu.tr; 7 Department of Animal Science, Faculty of Agriculture, Yozgat Bozok University, TR66000 Yozgat, Türkiye; hacer.tufekci@bozok.edu.tr; 8 Department of Animal Science, Faculty of Agriculture, Gaziosmanpaşa University, TR60250 Tokat, Türkiye; lutfi.bayyurt@gop.edu.tr; 9 Department of Food Processing, Vocational School of Ulubey, Ordu University, TR52850 Ulubey, Türkiye; hilalcelik@odu.edu.tr; 10 Department of Animal Science, Faculty of Agriculture, Ondokuz Mayis University, TR55139 Samsun, Türkiye; omer.yilmaz@omu.edu.tr

**Keywords:** FAMACHA©, anemia, support vector machine, machine learning, classification

## Abstract

The aim of this study is to evaluate the model performance in the classification of FAMACHA© scores using Support Vector Machines (SVMs) with a focus on the estimation of the FAMACHA© scoring system used for early diagnosis and treatment management of parasitic infections. FAMACHA© scores are a color-based visual assessment system used to determine parasite load in animals, and in this study, the accuracy of the model was investigated. The model’s accuracy rate was analyzed in detail with metrics such as sensitivity, specificity, and positive/negative predictive values. The results showed that the model had high sensitivity and specificity rates for class 1 and class 3, while the performance was relatively low for class 2. These findings not only demonstrate that SVM is an effective method for classifying FAMACHA© scores but also highlight the need for improvement for class 2. In particular, the high accuracy rate (97.26%) and high kappa value (0.9588) of the model indicate that SVM is a reliable tool for FAMACHA© score estimation. In conclusion, this study demonstrates the potential of SVM technology in veterinary epidemiology and provides important information for future applications. These results may contribute to efforts to improve scientific approaches for the management of parasitic infections.

## Simple Summary

In this study, the model performance in classifying FAMACHA© scores was evaluated using Support Vector Machines (SVMs) by focusing on estimating the FAMACHA© scoring system used in the early diagnosis and treatment management of parasitic infections. The reliability of the SVM model used in this study was examined in detail with metrics such as sensitivity, specificity, and positive/negative predictive values. As a result, it was revealed that SVM is an effective method in classifying FAMACHA© scores and provides important information for future applications. These results may contribute to the development of scientific approaches for the management of parasitic infections.

## 1. Introduction

Herd management in animal husbandry covers all kinds of practices, such as health, reproduction, etc., from the birth of an animal until its slaughter. In this context, sound planning is needed regarding herd management strategies for sustainable animal husbandry [1]. Therefore, herd management plans developed with an integrated approach play a fundamental role in improving not only production processes but also animal welfare.

The success of herd management strategies depends on the accurate and timely assessment of animal health. This assessment is essential for diagnosing and controlling common health problems such as parasite-related anemia. Parasites cause severe economic losses in small ruminants and negatively affect animal welfare [2].

In managing parasitic risks, biological parameters such as body condition score (BCS) and hematological blood traits provide important information in understanding animals’ general health status and parasite load. In this context, BCS was initially developed for sheep and dairy cattle [3,4]. BCS provides a practical tool for assessing animals’ energy reserves and general conditions [5]. Firstly, this process was proposed by Jefferies [6], and it was scaled from 0 to 5 [5]. The reasons for poor nutritional status in the herd include insufficient space per animal, problems in feed supply, endoparasite infestation, other chronic diseases, and problems related to herd management, such as dental problems [4,7]. In order to overcome these difficulties and develop more specific diagnostic methods, hematological parameters can be used to examine in detail the blood profile of each animal for conditions such as anemia and infection. The importance of such hematological assessments increases, especially when traditional clinical signs are insufficient to detect the burden of some parasites such as *Haemonchus* [8]. *Haemonchus* can cause serious health problems without showing obvious or early clinical signs. This situation emphasizes the need for more sensitive and specific diagnostic methods such as the FAMACHA© scoring system, which directly assesses anemia levels. Therefore, the integration of the FAMACHA© score with blood characteristics modeling in our study is particularly important in filling the gaps left by traditional CS-based diagnostic methods, and detailed analysis of hematological parameters contributes to sustainable animal husbandry practices by increasing the accuracy and reliability of these diagnostic processes. Detailed examination of hematological parameters is vital for the early diagnosis of certain diseases, and this, when combined with the FAMACHA© scoring, can provide reliability and accuracy, especially in the detection of anemia. In this context, anemia is critical for sustainable herd management. Especially during some physiological periods with high nutritional demand (e.g., lactation and gestation), ruminants may become more vulnerable to metabolic and endoparasite infestation disorders, and this is reflected in biochemical and hematological parameters [9]. Endoparasite infestations can be negatively affected by breeders’ income significantly.

**Figure 1:**
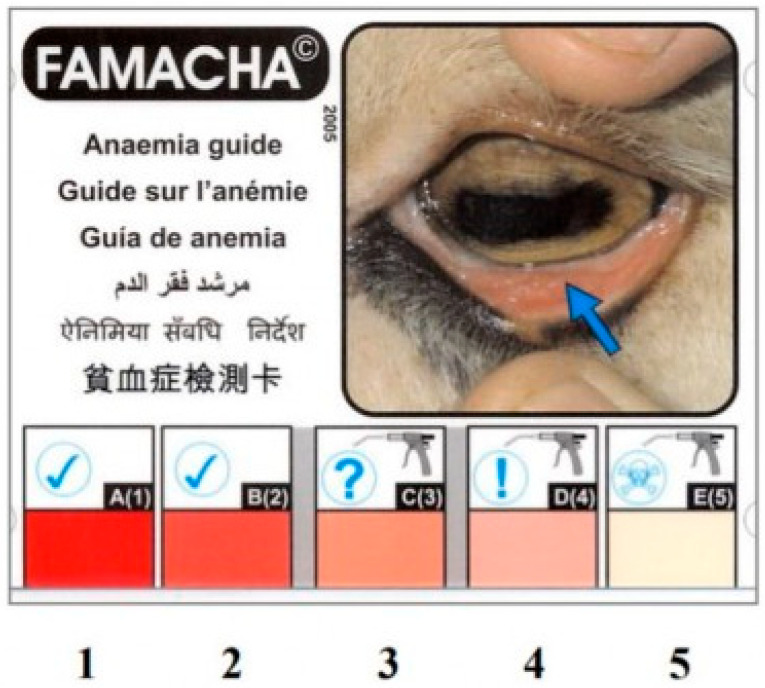
FAMACHA© card [12].

In this context, a need has arisen for a scoring system to evaluate anemia and indirectly detect parasitic infestations. The FAffa MAlan CHArt (FAMACHA©) scoring system was established for targeted selective treatment of sheep with hemonchosis-causing anemia in sheep [4,10]. The FAMACHA© scoring system is a practical and economical method used to determine the degree of anemia, especially in small ruminants. This system is based on color changes in the eye conjunctiva of animals [11]. The color of the conjunctiva provides information about the severity of anemia present in the animal. From this point of view, the FAMACHA© system is a beneficial tool for detecting clinical anemia [12]. This scoring system is from 1 to 5 (1: optimal; 2: acceptable; 3: borderline; 4: dangerous; 5: fatal) (In Figure 1) [4].

The current study aims to evaluate the success of the Support Vector Machine (SVM) algorithm in classifying FAMACHA© scores based on body condition score (BCS) and hematological parameters in Pelibuey sheep. Our research hypothesis is that the SVM algorithm can provide high sensitivity and specificity values in classifying the FAMACHA© scoring system used to determine parasite load, thus contributing to the development of early diagnosis and management strategies.

## 2. Materials and Methods

### 2.1. Study Area

The study was carried out at the commercial farm “El Rodeo”, located at 17°84′ N and 92°81′ W, approximately 14 km from the Villahermosa–Jalapa motorway in Tabasco, Mexico.

### 2.2. Animals and Blood Samples

This study evaluated a total of 247 Pelibuey sheep. A total of 177 lambs, 97 females and 80 males aged 3–4 months, and 70 non-pregnant and non-lactating adult sheep aged 2–3 years were included in our study. A blood sample was taken from each animal to determine hematological variables. The animals were also assessed for health status. Only animals without diseases of the respiratory system, genitourinary system, digestive system, musculoskeletal system, and nervous system were included.

In addition, parasitological analyses of fecal samples were performed on the same day as the clinical evaluation using previously described sedimentation and flotation techniques [12,13,14]. Animals with parasites were excluded from the study.

BCS was scored on a scale of 1 to 5 [15], with 1 representing very thin animals and 5 representing obese animals. BCS was assessed by an experienced person.

Blood samples were then collected from the jugular vein using tubes containing EDTA as an anticoagulant (Vacutainer; BD Biosciences, Franklin Lakes, NJ, USA). Blood samples were placed in a container on wet ice and transported to the laboratory. Finally, fecal samples were collected to confirm the absence of gastrointestinal parasites.

Analysis of blood samples within the scope of hematological parameters were measured using an automatic analyzer (VetAutoreadTM, IDEXX Laboratories, Westbrook, ME, USA). Blood smears were prepared and stained with Diff-QuickTM (Hycel, Zapopan, Jalisco, Mexico) to observe erythrocyte morphology and discard the presence of hemoparasites. The analyzed hematological parameters were as given below:

RBC: Red Blood Cell count; MCV: Mean Corpuscular Volume; RDW: Red Cell Distribution Width; RDWa: Red Cell Distribution Width absolute; HGB: Hemoglobin; MCH: Mean Corpuscular Hemoglobin; MCHC/MCHCL: Mean Corpuscular Hemoglobin Concentration/Mean Corpuscular Hemoglobin Content Level; PLT: Platelet count; PCT: Plateletcrit; MPV: Mean Platelet Volume; PDW: Platelet Distribution Width; WBC: White Blood Cell count; LYMF: Lymphocytes; GRAN: Granulocytes; MID: Mid-range (or median) cells.

### 2.3. Statistical Analysis

#### Support Vector Machine (SVM)

Support Vector Machine (SVM), proposed as a machine learning method, was developed by Vapnik [16] in the early 1990s on the basis of statistical learning theory [16]. While the statistical method used in modeling and prediction is called Support Vector Regression (SVR), the method used in classification is called support vector classification (SVC) [17,18,19,20,21]. The SVC has been designed as a tool to solve supervised learning classification problems because it has strong generalization properties [22,23]. SVC is first explained with basic principles for linearly separable cases. Then, kernel functions are discussed to construct nonlinear decision boundaries. Finally, slack variables are introduced to allow for training errors when noise is present in the data and when a complete separation of the two classes is not required [23]. In an SVM, the most appropriate hyperplane must be determined to maximize the model’s generalization ability [24]. Many kernel functions, such as linear, polynomial, gaussian, radial basis function, sigmoid, etc., were suggested for this task. The radial basis function was the most commonly used kernel function in these functions [24].

Various metrics are used to evaluate the model using values such as true positive rate (TP), false positive rate (FP), and false negative rate (FN). However, confusion matrix is needed to calculate factors such as true positive rate (TP), false positive rate (FP), and false negative rate (FN). These values cause the formation of a confusion matrix. The confusion matrix presented in Table 1 shows the results of the classification of data obtained using the FAMACHA© scoring system with the SVM model. The matrix contains the number of samples that the model classified as true positive (TP), true negative (TN), false positive (FP) and false negative (FN), which allows us to evaluate the performance of the model in terms of both sensitivity and specificity. The TP and TN values reflect the cases where our model made correct predictions, while the FP and FN values indicate the classification errors of the model. The analysis of this matrix is critical in determining the reliability and accuracy of our model in anemia diagnosis, especially for parasite burden assessments based on the FAMACHA© scoring system. The results obtained measure the effectiveness of the SVM model in terms of both theoretical and practical applications and thus contribute to the development of future parasite management strategies.

Several performance metrics, such as accuracy, precision, sensitivity, specificity, and F1 score, were used to evaluate the SVC algorithm’s classification capability from true positive (TP), false positive (FP), and false negative (FN) values in the equation below.

(1) $$Accuracy=\frac{TP+TN}{TP+TN+FP+FN}$$

(2) $$Precision=\frac{TP}{\left(TP+FP\right)}$$

(3) $$Sensitivity(TPR)=\frac{TP}{\left(TP+FN\right)}$$

(4) $$Specificity(TNR)=\frac{TN}{\left(TN+FP\right)}$$

(5) $$F1=\frac{2*Precision*Recall}{Precision+Recall}$$

In addition, kappa statistics can be used to evaluate the model’s accuracy to determine classification capability. In Table 2, the critical kappa values are given.

All statistical evaluations were performed by R software (version 2024.12.0) [25]. The descriptive statistics of the current study was performed by “psych” package in R software [26]. To visualize all possible relationships between explanatory and response variables, “GGally” package was used [27]. To divide the data set as train and test set, “caret” package was used [28]. For the Support Vector Machine algorithm, “e1071” package was used [29]. To visualize all analysis results, the “ggplot2” package was used [30]. To obtain the ROC curves, “pROC” package was used [31].

## 3. Results

Table 3 presents the mean values and standard deviations of various blood parameters according to FAMACHA© scores (1, 2 and 3).

According to Table 3, as the FAMACHA© score increases, changes are observed in some blood parameters. For example, BW is highest in animals with a FAMACHA© score of 3 with 27.93 ± 2.22 kg, and lowest in animals with a FAMACHA© score of 1 with 25.99 ± 5.12 kg. This shows that animals with higher FAMACHA© scores are generally heavier. When erythrocyte-related parameters such as HGB, MCV, and MCHC/MCHCL are examined, it is observed that these values decrease as the FAMACHA© score increases. In particular, the MCHC/MCHCL parameter decreases from 33.98 ± 6.29 in FAMACHA© 1 to 30.62 ± 1.09 in FAMACHA© 3, which may indicate that the oxygen-carrying capacity of erythrocytes decreases with the worsening of anemia. In terms of platelet parameters such as MPV and PDW, a decreasing trend is observed as the FAMACHA© score increases, indicating the negative effect of parasitic infection on platelet functions. Finally, when looking at the changes in WBC and its subgroups (LYMF, GRAN, MID), animals in FAMACHA© 1 had the highest white blood cell count with 18.14 ± 9.6 WBC, while these numbers were lower in FAMACHA© 2 and 3 with 11.3 ± 5.08 and 14.93 ± 8.6, respectively. This indicates that the immune system is suppressed in animals with high parasite load and white blood cell dynamics change in response to infection.

**Figure 2:**
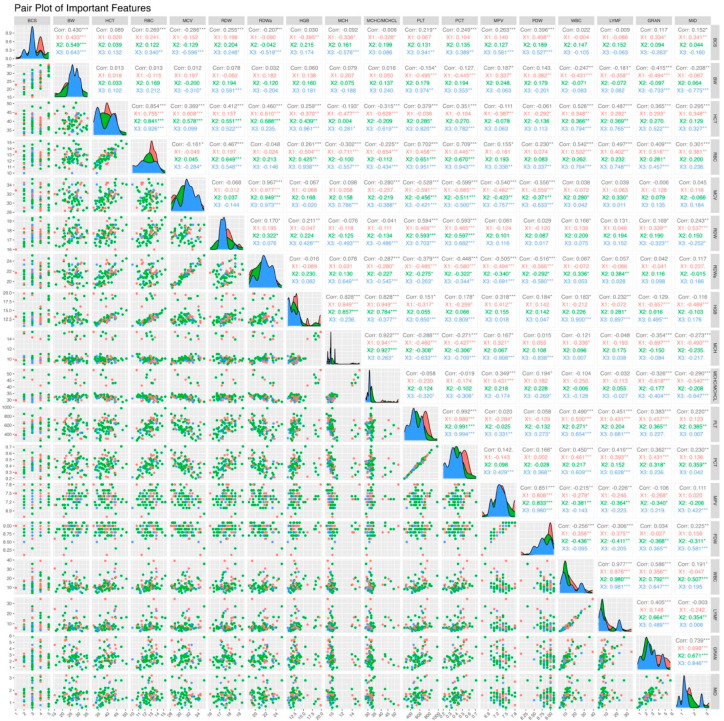
Correlation coefficients for each FAMACHA© scores. * *p* < 0.05, ** *p* < 0.01, *** *p* < 0.001.

Figure 2 is a pair of plots showing the correlations between selected important features based on FAMACHA© scores. This plot visualizes the relationships between each pair of features and shows how these features are distributed (scatter plots) and how they are distributed between each other (histograms). Both linear and non-linear relationships between features can be examined through these plots, making them an important tool in multivariate data analysis. In this image, each column and row represent a specific blood or biometric parameter, and each cell represents how these parameters relate to each other. Green dots represent a positive relationship, red dots represent a negative relationship, and the density and arrangement of the dots indicate the strength and character of the correlation. For example, a tight linear relationship can be observed in a cell where the dots are arranged along a straight line. Histograms show the frequency distribution of each feature, which can answer questions like whether the distribution of features is normal, skewed, or symmetrical. For example, whether a histogram is peaked or flat provides clues about how that feature varies in the dataset. This biplot matrix facilitates understanding of the relationships between traits and provides a basis for trait selection, modeling, or further statistical analysis. In particular, understanding how traits are related to each other for specific situations such as FAMACHA© scoring can help to better understand the effects of biological factors such as parasite load on animal health.

When general correlations are examined according to Figure 2, high positive correlations, such as 0.70 and 0.71, between RBC and HGB indicate that these two parameters increase together. This makes biological sense because hemoglobin is found in red blood cells and one of the basic functions of these cells is to carry oxygen. Therefore, an increase in the number of red blood cells usually leads to an increase in hemoglobin levels. On the other hand, an extremely high correlation of 0.99 between PLT and PCT indicates that these two measurements are closely related to each other. PCT expresses the platelet volume in the total blood volume and is naturally directly related to the platelet count. Interestingly, a moderate negative correlation of −0.56 between MCV and RBC generally indicates that the average volume of the cells decreases as the number of red blood cells increases. This is a phenomenon generally observed in cases of chronic anemia. However, a high correlation of 0.885 between RDWa and RDW suggests that these two parameters provide similar biological information, and both measurements are used to quantify variations in the size of red blood cells.

When the correlation matrices specific to FAMACHA© scores are examined, it is seen that the relationships between various biological parameters vary with FAMACHA© scores. These variations provide important information about how hematological parameters and biometric measurements can be affected by differences in parasitic load or health status. For example, when comparing low FAMACHA© scores (1 and 2) with high scores (3), it is noticeable that the correlations in parameters such as MCV (Mean Red Blood Cell Volume) and RDWa (Adjusted Red Blood Cell Distribution Width) differ. While very high correlations are observed between these parameters at low scores, this relationship weakens at score 3. This may indicate that animals with higher FAMACHA© scores may be characterized by increased levels of anemia and variable cell sizes, which may be associated with increased parasite load or exacerbation of disease status. Correlations between hemoglobin (HGB), mean corpuscular hemoglobin (MCH), and mean corpuscular hemoglobin concentration (MCHC/MCHCL) also vary according to FAMACHA© scores. While there are strong correlations between these parameters at lower scores, these correlations weaken at score 3. This suggests that advanced anemia states affect hemoglobin levels and intracellular hemoglobin concentration differently. Furthermore, correlations between immunological parameters such as white blood cells (WBCs), lymphocytes (LYMFs), and granulocytes (GRANs) also vary as FAMACHA© scores increase. Higher scores indicate decreased correlations, especially between lymphocytes and granulocytes, which may reflect the effect of different FAMACHA© scores on the immune system.

According to Table 4, it is shown that the classification model used to predict FAMACHA© scores has a high accuracy (97.26%) and effectiveness. The model was determined to have a high accuracy rate with a 95% confidence interval (0.9045, 0.9967), and the kappa statistic (0.9588) showed that the model was significantly superior to random guesses. This confirms that the classification performance of the model is statistically strong and reliable. The sensitivity, specificity, and prediction values of the model vary according to the classes. In particular, 100% sensitivity values for class 2 and class 3 indicate that all positive cases in these classes were successfully detected, while the sensitivity rate for class 1 was slightly lower (91.67%). While the specificity values were 100% for class 1 and class 3, they were determined as 96.08% for class 2. This indicates that there may be some false positive results in class 2. Positive predictive values (PPVs) and negative predictive values (NPVs) reflect the model’s ability to predict true positive and true negative. While PPV is 100% for class 1 and class 3, this rate is 91.67% for class 2. NPVs are high for all classes (between 96.08% and 100%), indicating that the model has a strong ability to correctly detect negative cases. Balanced accuracy values measure the degree to which the model predicts both classes (positive and negative) in a balanced manner. These values were determined as 95.83% for class 1, 98.04% for class 2, and 100% for class 3, indicating that the model exhibits an excellent balanced performance for class 3 while class 2 provides a very high balanced accuracy. In general, the high accuracy, sensitivity, and specificity values of this model and its effective predictive ability indicate that it can be a powerful tool in disease detection and management based on FAMACHA© scores. In addition, the F1 score for FAMACHA© 1 score was calculated as 0.956, FAMACHA© 2 score as 0.956, and FAMACHA© 3 score as 1.000. These results show that the model provides an excellent balance of sensitivity and specificity, especially in the FAMACHA© 3 class, indicating that the model performs flawlessly in distinguishing both positive and negative cases in this class. The high F1 scores for the other two classes also show that the model works with high accuracy and sensitivity in these classes and produces reliable results in general.

**Figure 3:**
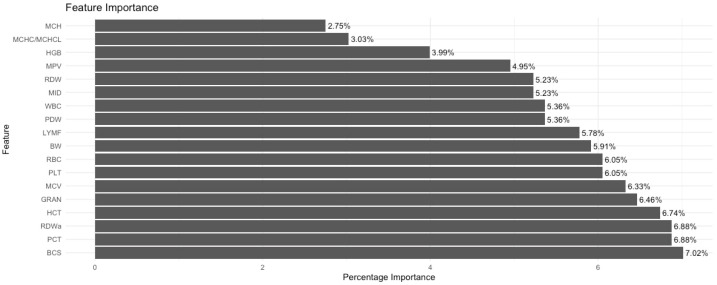
Variable importance values.

Figure 3 shows the variable importance levels, where the order of importance of the features in the models can be clearly observed. The graph is presented in a bar chart format, where the features are ranked according to their percentage importance.

According to Figure 3, the BCS trait is at the top with a rate of 7.02%. This trait has the highest importance in the model and has a great impact on the prediction performance. RDWa and PCT are in second place with 6.88%, which shows that these traits have a significant impact on the predictions in the model. The other important traits that continue in the list are HCT (6.74%), GRAN (6.46%), and MCV (6.33%), respectively. These traits are other main factors that significantly affect the performance of the model. Among the lower ranked traits, features such as MCH, MCHC/MCHCL, and HGB also contribute to the prediction capacity of the models, but they have a lower impact than those in the upper ranks. In particular, MCH (2.75%) and MCHC/MCHCL (3.03%) are shown as the least important traits, and their effects on the model remain relatively low.

**Figure 4:**
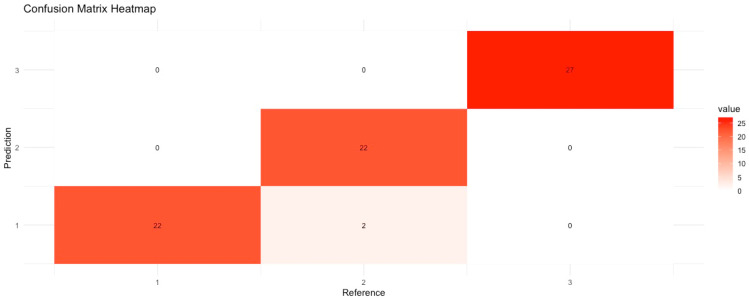
Confusion matrix for Support Vector Machine algorithm.

Figure 4 shows a heat map of the confusion matrix obtained using the Support Vector Machine algorithm. This heat map visually shows how successfully the algorithm classifies different classes and the possible misclassifications.

In the confusion matrix in Figure 4, the columns represent the predicted classes, and the rows represent the reference classes. The heat map shows the color intensity and the number of predictions in each matrix cell. Darker colors indicate higher values. First, for class 1, the model correctly recognized 22 cases as class 1. For class 2, the model correctly recognized 22 cases as class 2. However, the model also classified 2 cases as class 1. This indicates that class 2 is more likely to be confused with other classes. Finally, for class 3, the model perfectly classified class 3 and did not make any misclassifications in this class. The model appears to be quite successful in class 1 and class 3 predictions, but there is some confusion for class 2.

**Figure 5:**
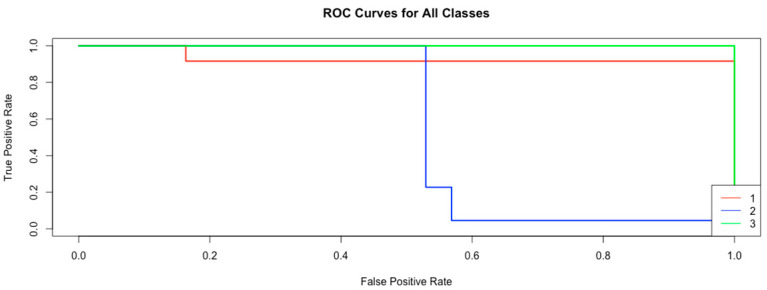
ROC curve for Support Vector Machine algorithm.

Figure 5 shows the Per Class ROC (Receiver Operating Characteristic) curves obtained using the Support Vector Machine (SVM) algorithm. ROC curves are used to evaluate the performance of the model at different classification thresholds; here the true positive rate (TP) is plotted against the false positive rate (FP).

According to Figure 5, the following results are obtained:

- Red Curve (Class 1): This curve again shows high performance; TPR value is very close to 1 while FPR value is very close to 0. This shows that the model is also very successful in recognizing class 1.
- Blue Curve (Class 2): This curve shows lower performance compared to the others. FPR jumps at a certain point, indicating that the model mistakenly classified some negative examples in this class as positive. However, TPR value eventually reaches 1, indicating that all positive cases are finally classified correctly.
- Green Curve (Class 3): This curve shows an almost perfect performance for class 3. FPR is almost 0 and TPR is almost 1. This shows that the model recognizes class 3 with little to no errors and makes a high-accuracy classification with very few false positives.

In Figure 5, overall, these ROC curves show that the model can recognize class 3 and class 1 very well, but its performance on class 2 is noticeably lower.

The current study on the prediction of FAMACHA© scores using Support Vector Machines (SVMs) has shown that this method can be an effective tool in parasite load assessment. According to the analysis results, the SVM model made successful predictions with very high accuracy rates, especially for class 1 and class 3. The sensitivity and specificity rates for both classes reached almost perfect levels, and almost no false positive predictions were made in these classes. The model showed a slightly lower performance for class 2, and areas for improvement were determined in this class.

In general, the high accuracy rate (97.26%) and kappa statistic (0.9588) obtained prove that SVM is a reliable and effective method in classifying FAMACHA© scores. These results provide an important reference point, especially in the early diagnosis of parasitic infections and in determining the relevant treatment strategies. However, the difficulties in class 2 classification indicate that the model needs to be further optimized for this particular class. This study provides a solid foundation for the potential applications of SVM in the field of veterinary epidemiology and provides avenues for expanding this technology.

## 4. Discussion

This study discusses the classification of visual FAMACHA© scores in Pelibuey sheep using the Support Vector Machine (SVM) algorithm. Our study provides significant findings on how body condition score and hematological parameters can effectively determine parasite burden and early diagnosis management. The success of the SVM algorithm in classifying FAMACHA© scores with high accuracy, sensitivity, and specificity rates highlights the potential applications of this method in veterinary epidemiology. However, the relatively low performance of our model in class 2 predictions indicates that the algorithm needs to be further improved for this particular class. In this context, in our discussion section, we will discuss potential strategies that can increase the classification success of the SVM algorithm and the effects of this algorithm on sheep health management.

Currently, studies using the SVM algorithm to classify FAMACHA© scores are quite limited. Other studies on anemia classification are presented below. The anemia detection method developed by Hermoza et al. [32] achieved 79% sensitivity and 91% specificity values when classifying the probability of anemia as high, moderate, or low using photographic images of the patient’s nail bed. These results show the potential of using the anemia semaphore as a screening method that can reduce the need for blood tests. In comparison, the FAMACHA© scoring performed with the Support Vector Machine (SVM) algorithm used in our study showed higher performance with 100% sensitivity and specificity rates, especially for class 1 and class 3. However, a lower rate of 96.08% specificity was obtained for class 2 compared to Hermoza’s method, indicating that the SVM algorithm may produce false positive results in this class and may require improvement. These differences require evaluation of both methods in terms of specific usage scenarios and improvement needs.

In the study of Mohammed et al. [33], the rule-based classification techniques applied reached an accuracy rate of 85% with the PART method. These techniques were used to determine the basic rules in the dataset and to provide a basis for further research. In contrast, the Support Vector Machine (SVM) algorithm used in the current study provided 100% sensitivity and specificity values for FAMACHA© scoring in class 1 and class 3, and 100% sensitivity and 96.08% specificity in class 2. These results show that the SVM algorithm provides a higher accuracy rate compared to rule-based techniques, especially for class 1 and class 3. However, the lower specificity rate in class 2 indicates that the model needs to be improved for this class.

In the study of Siddique et al. [34], an artificial intelligence-supported biosensor was tested on blood samples taken from small ruminants. While this biosensor initially detected anemia with an accuracy rate of 76.06%, this rate increased to 95.8% with the improvement of model parameters. This study, which was conducted using various machine learning models such as SVM, KNN, BPNN, and Keras, analyzed blood samples and successfully classified PCV ranges from healthy to severe anemic conditions with an F1 score of 74–100%. On the other hand, the Support Vector Machine (SVM) algorithm that we used in our current study reached 100% sensitivity and specificity rates in the detection of anemia carried out via FAMACHA© scoring, which revealed that SVM is extremely effective in determining high-risk conditions. When the study of Siddique et al. [33] and our own study were compared, it was seen that both methodologies showed high success in the detection of anemia. However, the SVM algorithm appears to offer unique advantages in terms of accuracy and reliability, especially in challenging agricultural environments and in the context of animal health management. These results demonstrate how AI-enabled technologies can be effective in animal health monitoring and disease management applications.

The study conducted by Kaplan et al. [35] evaluated the usability of the FAMACHA© method in the detection of anemia caused by *Haemonchus contortus* and revealed that it showed high sensitivity but had some limitations in terms of specificity. In particular, the sensitivity was 100% when the PCV value determined for anemia was ≤15, but this criterion resulted in low specificity that could cause some healthy animals to be incorrectly labeled as anemic. This could lead to unnecessary treatment of animals that do not need treatment, which increases drug costs and may accelerate the development of anthelmintic resistance. In our current study, 100% sensitivity and specificity were achieved in the detection of anemia carried out via FAMACHA© scoring using the Support Vector Machine (SVM) algorithm. These results show that the SVM algorithm offers unique advantages in terms of accuracy and reliability, especially in difficult agricultural environments and in the context of animal health management. The high sensitivity and specificity rates indicate that anemia detection using SVM maximizes the capacity to make correct diagnoses while minimizing false positive and negative rates. This comparative analysis may allow us to better understand how they can play a role in integrated parasite control strategies, considering the advantages and limitations of both methods.

In our study, high sensitivity and specificity values were achieved in the detection of anemia with the SVM algorithm using the FAMACHA© scoring. These findings show that the SVM algorithm offers unique advantages in terms of accuracy and reliability, especially in difficult agricultural environments and in the context of animal health management. The current study, when compared to other studies in the literature, reveals that the SVM algorithm is an effective tool in determining high-risk situations and detecting anemia. It proves how effective artificial intelligence-supported technologies can be in animal health monitoring and disease management applications. In this context, these studies, which provide a better understanding of how SVM-based methods can play a role in integrated parasite control strategies, will constitute an important reference point for future research. Therefore, it is of great importance to determine strategies for the optimization of the integration of methods such as FAMACHA© and SVM in the fight against anthelmintic resistance and in the development of sustainable parasite control programs.

## 5. Conclusions

This study addresses the usability of the Support Vector Machines (SVMs) method in the assessment of parasite load in animals via the FAMACHA© scoring system. The SVM model showed impressive performance with high accuracy, sensitivity, and specificity values, especially for the first and third classes. However, the relatively low performance observed in the second class compared to other groups indicates that the model needs to be further improved in this particular area. This finding is a critical issue that should be addressed as a priority during the model development process.

The results of the study show that the SVM method has significant potential in correctly classifying and managing parasite load. These results may be of great importance when developing early diagnosis and treatment strategies for parasitic infections. In particular, this technique is a valuable tool that will help veterinarians optimize parasite control strategies.

However, the scope of the study needs to be expanded in order to increase the generalizability of the model and to test its applicability to other sheep breeds or large farm animal populations. Such an expansion will provide the opportunity to evaluate the performance of the model under different genetic structures and geographical conditions.

To obtain clearer information about the limitations of the study, the model needs to be tested on larger and more diverse datasets. This has the potential to increase the robustness of the model and reduce misclassification rates. In addition, a more in-depth analysis of the features on which the model is based will increase its interpretability and biological validity. This study is believed to provide a solid foundation for further research in this area, while encouraging the integration of machine learning approaches for the management of parasite load.

## Figures and Tables

**Table 1:** Confusion matrix.

Forecast	Reality	
	Positive	Negative
Positive	TP	FP
Negative	FN	TN

**Table 2:** Relationship between classification accuracy and kappa coefficient.

Accuracy of the Classification	Kappa Coefficient
Very poor	<0
Poor	0.00–0.20
Average	0.20–0.40
Good	0.40–0.60
Very good	0.60–0.80
Excellent	0.80–1.00

**Table 3:** Descriptive statistics according to each FAMACHA© score.

	FAMACHA©		
	1	2	3
N	81	75	91
BW	25.99 ± 5.12	26.28 ± 4.72	27.93 ± 2.22
HCT	40.16 ± 3.26	39.33 ± 4.26	39.55 ± 4.37
RBC	12.86 ± 0.91	12.51 ± 1.23	12.37 ± 1.38
MCV	31.14 ± 1.96	31.45 ± 1.89	31.97 ± 1.37
RDW	17.8 ± 0.6	17.44 ± 0.9	17.53 ± 0.46
RDWa	21.35 ± 1.53	21.41 ± 1.44	21.92 ± 1.19
HGB	13.6 ± 2.37	12.91 ± 1.93	12.08 ± 1.18
MCH	10.47 ± 1.95	10.31 ± 1.26	9.77 ± 0.42
MCHC/MCHCL	33.98 ± 6.29	32.94 ± 4.24	30.62 ± 1.09
PLT	614.72 ± 151	542.49 ± 144.06	517.38 ± 121.19
PCT	0.46 ± 0.11	0.4 ± 0.11	0.38 ± 0.09
MPV	7.38 ± 0.25	7.31 ± 0.24	7.26 ± 0.15
PDW	8.87 ± 0.22	8.85 ± 0.2	8.78 ± 0.17
WBC	18.14 ± 9.6	11.3 ± 5.08	14.93 ± 8.6
LYMF	13.09 ± 9.37	7.38 ± 4.15	10.77 ± 7.93
GRAN	3.31 ± 1.3	2.34 ± 0.99	2.74 ± 1.08
MID	1.74 ± 0.62	1.58 ± 0.61	1.42 ± 0.54

RBC: Red Blood Cell count; MCV: Mean Corpuscular Volume; RDW: Red Cell Distribution Width; RDWa: Red Cell Distribution Width absolute; HGB: Hemoglobin; MCH: Mean Corpuscular Hemoglobin; MCHC/MCHCL: Mean Corpuscular Hemoglobin Concentration/Mean Corpuscular Hemoglobin Content Level; PLT: Platelet count; PCT: Plateletcrit; MPV: Mean Platelet Volume; PDW: Platelet Distribution Width; WBC: White Blood Cell count; LYMF: Lymphocytes; GRAN: Granulocytes; MID: Mid-range (or median) cells.

**Table 4:** Performance metrics of FAMACHA© scoring results with Support Vector Machine.

Statistic					Value				
Accuracy					0.9726				
95% CI					(0.9045, 0.9967)				
No Information Rate					0.3699				
*p*-Value [Acc > NIR]					<2.2 × 10^−16^				
Kappa					0.9588				
FAMACHA© score	Sensitivity	Specificity	Pos Pred Value	Neg Pred Value	Prevalence	Detection Rate	Detection Prevalence	Balanced Accuracy	F1 score
1	0.916	1.0000	1.0000	0.9608	0.3288	0.3014	0.3014	0.9583	0.956
2	1.000	0.9608	0.9167	1.0000	0.3014	0.3014	0.3288	0.9804	0.956
3	1.000	1.000	1.0000	1.0000	0.3699	0.3699	0.3699	1.0000	1.000

## Data Availability

The data presented in this study are available on request from the corresponding author.
